# Expression and prognostic analyses of SCAMPs in pancreatic adenocarcinoma

**DOI:** 10.18632/aging.202377

**Published:** 2021-01-20

**Authors:** Feiyu Mao, Heng Duan, Aly Allamyradov, Zechang Xin, Yan Du, Xiaodong Wang, Peng Xu, Zhennan Li, Jianjun Qian, Jie Yao

**Affiliations:** 1 Clinical Medical College of Yangzhou University, Yangzhou 225001, Jiangsu Province, China; 2 The First Affiliated Hospital of Dalian Medical University, Dalian 116044, Liaoning Province, China; 3 Department of Hepatobiliary and Pancreatic Surgery, Northern Jiangsu People’s Hospital, Guangling Qu, Yangzhou 225001, Jiangsu Province, China

**Keywords:** SCAMPs, differential expression, prognostic biomarkers, pancreatic adenocarcinoma

## Abstract

Due to the difficulties in early diagnosis of pancreatic adenocarcinoma (PAAD), many patients fail to receive optimal therapeutic regimens. The Secretory-Carrier-Membrane-Proteins (SCAMPs) are known to be dysregulated in a range of human diseases due to their characterized roles in mammalian cell exocytosis inferred from their functions as integral membrane proteins. However, the expression and prognostic value of SCAMPs in PAAD is poorly characterized. We compared cancer vs. healthy tissue and found that the expression of SCAMPs1-4 was upregulated in PAAD compared to normal tissue. In contrast, SCAMP5 expression was downregulated in PAAD. Moreover, the expression of SCAMPs1-4 was enhanced in PAAD cell lines according to Cancer Cell Line public database. Furthermore, the HPA, GEPIA databases and immunohistochemical analysis from 238 patients suggested that the loss of SCAMP1 led to improved overall survival (OS), whilst lower SCAMP5 levels led to a poorer OS. The univariate and multivariate analysis showed that SCAMP1 and SCAMP5 expression were independent prognostic factors of PAAD. In addition, the cBioPortal for Cancer Genomics, LinkedOmics datasets, and the GEPIA were used to identify the co-expression genes of SCAMP1,5 and the correlation between SCAMPs members. We conclude that SCAMPs 1 and 5 significantly represent promising diagnosis and prognostic biomarkers.

## INTRODUCTION

Pancreatic cancer remains a major cause of cancer related death. Pancreatic tumors develop from the dysregulated proliferation of cells in the pancreas. Approximately 50% of cases of pancreatic cancer are diagnosed in those aged over 75. The most common type of pancreatic cancer is pancreatic ductal adenocarcinoma, the current therapies for which including surgery, chemotherapy and radiotherapy are ineffective. Due to the difficulties of treating malignant PAAD tumors, many patients have failed to receive optimal therapeutic regimens. New and more effective diagnosis and treatments for this and other forms of pancreatic cancer are therefore urgently required.

SCAMPs function as post-Golgi transporters in all mammalian cells. The expression of the SCAMP family members differs in many cell types, with each SCAMP proposed to act during key stages of post-Golgi transport [1]. SCAMPs 1-3 possess a cytoplasmic N-terminal domain with multiple NPF (R-P-F) repeats, conserved transmembrane regions (TMs) and a cytoplasmic tail that mediates surface to Golgi transport [2]. However, SCAMPs 4-5 differ from other family members as they lack a highly conserved cytoplasmic NPF repeat [3]. SCAMPs are integral membrane proteins that are ubiquitously expressed, most notably in secretory cells [4–6]. Although SCAMP functionality is yet to be defined, previous studies highlight their role in endocytosis, exocytosis and vesicular trafficking [7–9]. SCAMPs co-exist in circulating transport vesicles originating from post-Golgi and endocytic transport and so do not function during endosome recycling. SCAMPs therefore act at similar stages of post-Golgi function though the precise functionality of each SCAMP member remains poorly defined [9].

SCAMP1 dampens down the invasive ability of MTSS1 in triple-negative breast cancer cells through the trafficking mediated upregulation of RAC1-GTP, thus enhancing cell adhesion. The cooperative activity of MTSS1 and SCAMP1 prevents triple-negative breast cancer cell invasion whilst their silencing enhances the aggressiveness of these cancer cells [10]. We previously demonstrated that SCAMP1 silencing inhibits the metastatic phenotypes of human pancreatic and gallbladder cancer cells [11]. SCAMP1 is differentially expressed in normal vs. tumor tissue in patients with cervical cancer and pancreatic cancer lacking lymph node metastasis [11, 12].

The growth of melanoma tumors is inhibited following EFEMP1 and SCAMP3 silencing by miR-192-5p and miR-584-3p targeting, respectively. The AMPK activator Metformin also displays anti-cancer activity, particularly in cases of melanoma in which cancer cell growth is inhibited through direct effects on miR-192-5p-EFEMP1 and miR-584-3p-SCAMP3 pathways [13]. SCAMP3 also mediates inflammatory responses in breast cancer cells [14]. SCAMP4 accumulates on the surface of senescent cells and promotes SASP (The senescence-associated secretory phenotype) factor secretion, in addition to IL6, IL8, and growth differentiation factor 15 (GDF-15). Moreover, SCAMP3 promotes an SASP phenotype, a major trait of senescent cells [4]. However, knowledge of the cellular roles of SCAMP5 are limited in comparison to other family members. Unlike other SCAMP family members, neuronal SCAMP5 contributes to endocytic recycling to promote neuronal conduction [15].

In this study, we assessed cancer vs. healthy tissue and found that the expression of SCAMPs1-4 is higher in PAAD compared to normal tissue. In contrast SCAMP5 expression was downregulated in PAAD. Moreover, the expression of SCAMPs 1-4 were enhanced in *in vitro* PAAD cell lines according to online cancer databases. Our immunohistochemical analysis and HPA, GEPIA databases were used to measure the differential expression of SCAMPs and survival analysis based on HPA, GEPIA databases which suggested that the lower SCAMP1 led to improved overall survival (OS), whilst the low levels of SCAMP5 led to a poor OS. In addition, the cBioPortal for Cancer Genomics, LinkedOmics datasets and GEPIA were used to analyze the correlation between SCAMPs 1, 4 and 5 in PAAD in which a significant correlation was identified. These data highlight SCAMPs as important diagnostic and therapeutic targets for PAAD.

## RESULTS

### Differential expression and diagnosis model of SCAMPs mRNA between PAAD and normal samples

**Figure 1:**
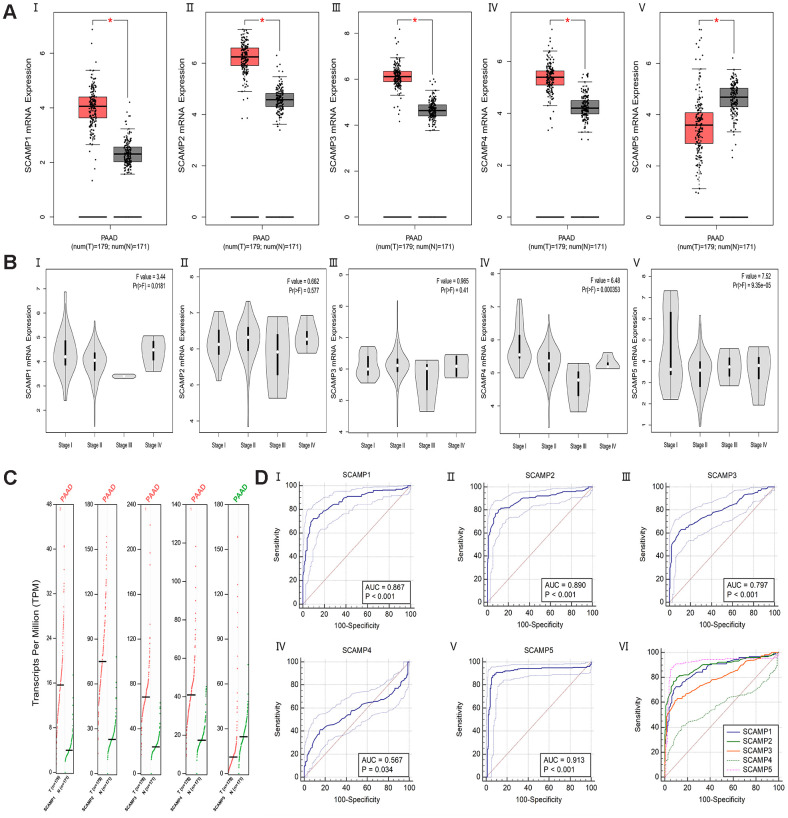
**Differential expression and ROC curves of SCAMPs in PAAD.** (**A**) Differential expression of SCAMP 1-5 (I-V) in PAAD (log2(TPM + 1)). (**B**) Expression violin plots of SCAMP 1-5 (I-V) based on patient pathological stage (log2(TPM + 1)). (**C**) Differential expression of SCAMP 1-5 in PAAD (TPM). (**D**) The Area Under the Curve (AUC) metrics are also provided for SCAMP1-5 (I-V) to predict diagnosis in PAAD by Medcalc (version 19.0); the comparison of ROC curves for SCAMP1-5 (VI).

According to the Gene Expression Profiling Interactive Analysis (GEPIA) datasets, an Online web-analysis tool frequently used to assess TCGA and GTEx databases, mRNA profiles of individual SCAMPs were compared between normal tissue and PAAD. The results showed that SCAMPs 1-4 were expressed to higher levels in PAAD than normal samples, whilst the opposite phenotype was observed for SCAMP5 (Figure 1A, 1C). In addition, expression violin plots showed SCAMP1 and SCAMP5 had a significant correlation with patients’ pathological stage (Figure 1B). Next, ROC curves indicated that the AUC index in TCGA and GTEx datasets was 0.867(p<0.001), 0.890(p<0.001), 0.797(p<0.001), 0.567(p<0.05) and 0.913(p<0.001) respectively (Figure 1D). Additionally, we also found the significant difference in SCAMP1 (p < 0.05) and SCAMP5 (p < 0.05) between early stage (Stage I + II) and late stages (Stage III + IV) as diagnosis markers. (Supplementary Figure 1A, 1B) Because the sample size of PAAD in Stage III + IV is small, we also calculated the difference in SCAMP1 (p > 0.05) and SCAMP5 (p < 0.05) between early stages (Stage I + II a) and late stages (Stage II b + III + IV) (Supplementary Figure 1C, 1D).

### Expression of SCAMPs in PAAD cells

**Figure 2:**
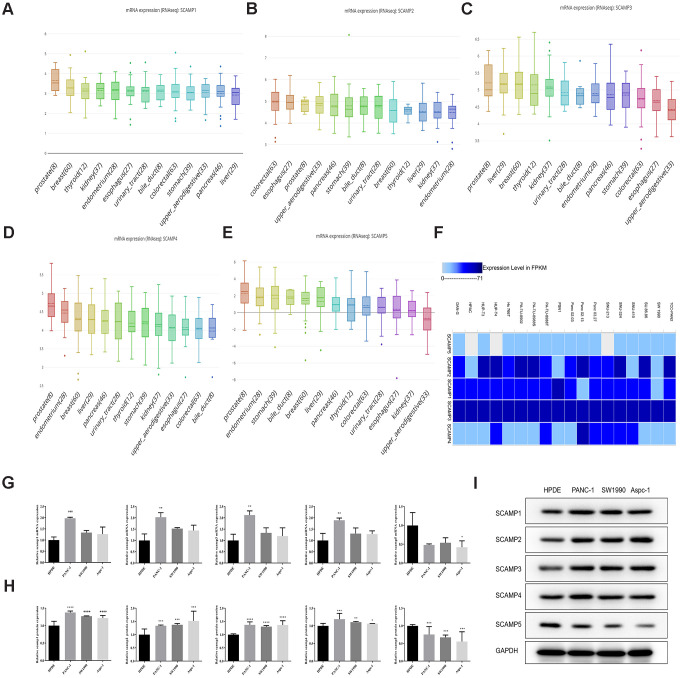
**Expression of SCAMPs in PAAD cell lines using CCLE and the EMBL-EBI: Expression Atlas.** (**A**–**E**) Expression of SCAMPs in PAAD cell lines using the CCLE database. (**F**) Expression of SCAMPs in PAAD cell lines using the EMBL-EBI (Expression Atlas) database. (**G**–**I**) The verification of SCAMPs expression was evaluated by RT-qPCR (**G**) and western blotting (**H**, **I**) in PAAD cell lines (PANC-1, SW1990 and AsPC-1 cell) and normal human pancreatic ductal cell (HPDE cell). Results shown are the mean ± SD (* *p* < 0.05, ** *p* < 0.01, *** *p* < 0.001).

Using the Cancer Cell Line Encyclopedia (CCLE) database and the bubble heatmap visualizer derived from the GTEx-Portal, gene expression data was visualized from the datasets. Upon the assessment of an array of cancer cell lines, SCAMPs 1-4 were highly expressed, but SCAMP5 was expressed to lower levels in PAAD cells (Figure 2A–2E). The European Bioinformatics Institute (EMBL-EBI) database was also used to further assess SCAMP expression in the PAAD cells. The analysis confirmed that SCAMPs1-4 were overexpressed in the majority of PAAD lines, whilst SCAMP5 was expressed to low levels (Figure 2F).

We also validated the expression of SCAMPs in PAAD cell lines (PANC-1, SW1990 and AsPC-1 cell) and normal human pancreatic ductal cell (HPDE cell) by Real-time quantitative PCR and Western blot assay. The result showed that SCAMPs1-4 were overexpressed in PANC-1 cell; whilst SCAMP5 was expressed to low levels in AsPC-1 cell line (Figure 2G–2I).

### Prognostic analysis of the SCAMPS in PAAD

**Figure 3:**
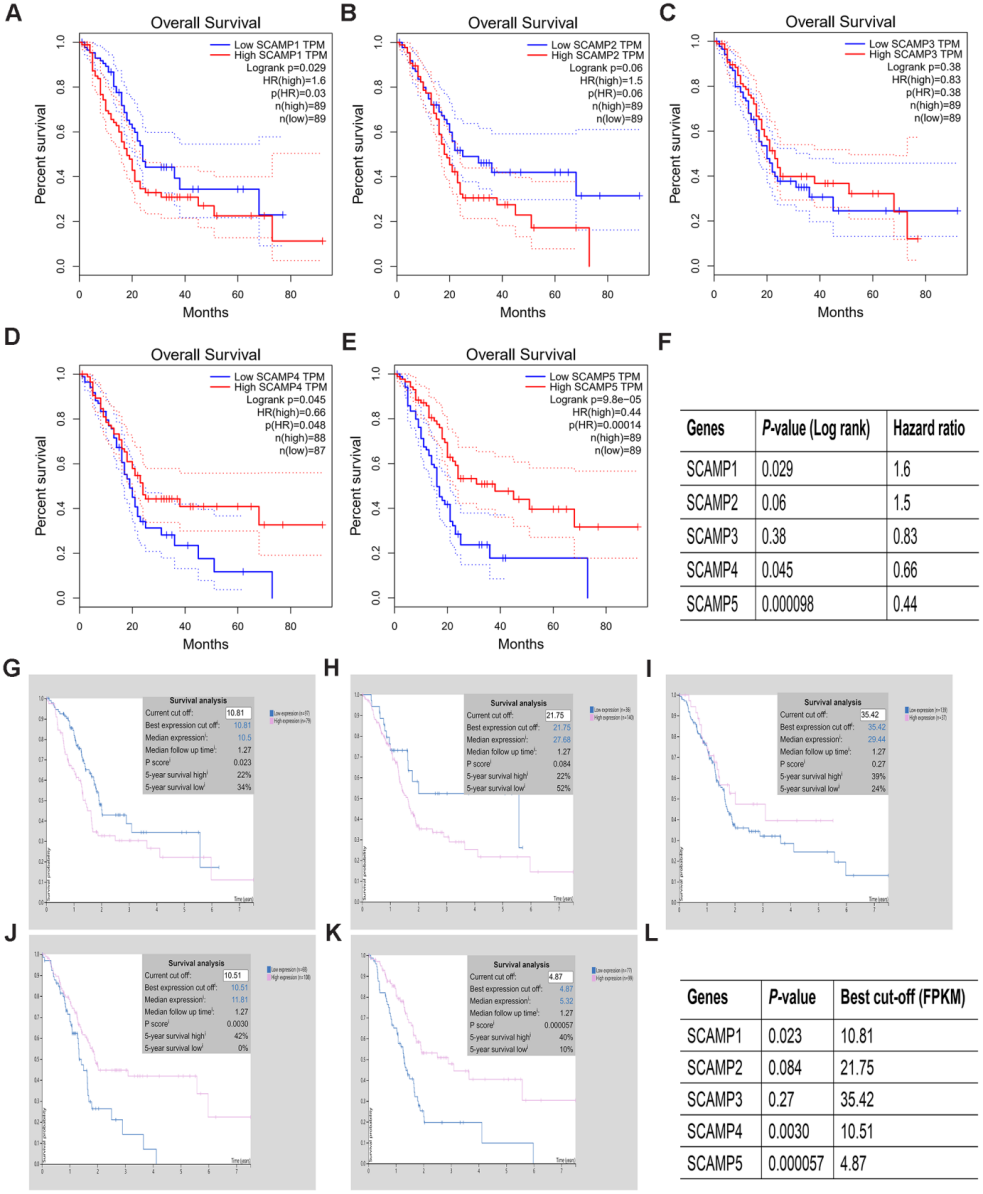
**Prognostic value of SCAMP expression in PAAD patients (HPA and GEPIA).** (**A**–**L**). Prognostic value of SCAMP expression in PAAD patients according to GEPIA (**A**–**F**) and HPA (**G**–**L**) databases.

We next performed survival analysis for SCAMPs 1-5 using the Human Protein Atlas (HPA) and GEPIA databases in PAAD. The data showed that SCAMPs 1,5 were strongly associated with a poor OS in PAAD from both databases (Figure 3A, 3B). SCAMP5 downregulation was associated with poor OS in PAAD (Figure 3A, 3B) whilst SCAMP1 overexpression led to a poor prognosis, suggesting its role as an oncogene. Thus, both SCAMPs 1, 5 act as predictors for the diagnosis and prognosis of PAAD.

### SCAMP1 and SCAMP5 expression and their correlation with the clinicopathological characteristics and prognosis of PAAD

**Figure 4:**
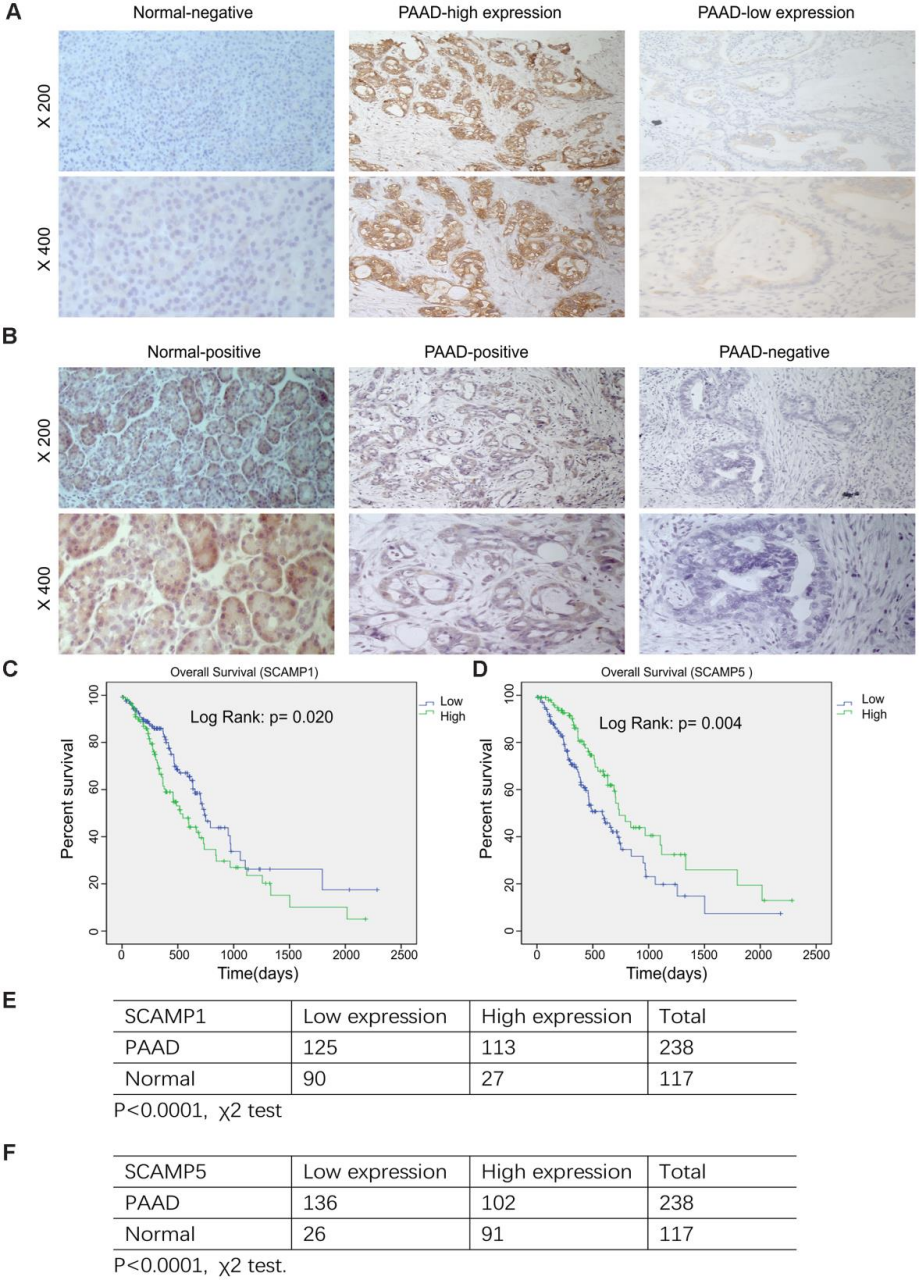
**Relationship SCAMP1, 5 expression and the clinicopathological parameters of PAAD patients.** (**A**, **B**) Representative images of SCAMP1 and SCAMP5 staining in PAAD tissue. (Expression of SCAMP1 and SCAMP5 were evaluated semi-quantitatively based on staining intensity and cell positivity, representative images are shown at × 200 and × 400 magnification, respectively.) (**C**, **D**) Kaplan–Meier analysis of the overall survival of PAAD patients stratified by the SCAMP1 and SCAMP5 immunoreactive scores by SPSS version 19.0. Log-rank test were performed to compare differences between groups. (**E**, **F**) Quantification of SCAMP1, 5 expression in pancreatic cancer and adjacent normal samples. Statistical analyses were performed using the χ2 test. Low: low expression, High: high expression.

We used immunohistochemistry to assess the expression of SCAMP 1, 5 in 238 paraffin-embedded PAAD and 117 adjacent normal specimens. Immunostaining for SCAMP1,5 was mostly cytoplasmic/membranous in PAAD cells (Figure 4A, 4B). Upon analysis of SCAMP1,5 expression with clinicopathologic parameters, SCAMP1 overexpression positively correlated with age, N stage, TNM stage and Neural invasion, whilst the down-regulation of SCAMP5 correlated with age, T stage, N stage, TNM stage and pathology differentiation (Table 1). Kaplan–Meier survival plots showed that higher SCAMP1 or lower SCAMP5 expression correlated with poorer prognosis in PAAD patients (log-rank test, *p* *=0.020; p=0.004*, Figure 4C, 4D). Moreover, univariate analysis indicated that age, metastasis, SCAMP1 expression and SCAMP5 expression were significantly associated with the risk of cancer-related death. Multivariate analysis showed that SCAMP1 and SCAMP5 expression were independent prognostic factors (Table 2). Chi-Square tests were used to investigate SCAMP1, 5 expression in adjacent normal and pancreatic adenocarcinoma (p<0.0001), (Figure 4E, 4F).

**Table 1:** Relationship between SCAMP1, 5 expression and the clinicopathological parameters of patients with PAAD.

**Characteristics**	**Number of cases**	**SCAMP1 level**			**SCAMP5 level**		
		**L**	**H**	**P value**	**L**	**H**	**P value**
Total cases	238						
Sex							
Male	128	64	64	0.401	74	54	0.822
Female	110	61	49		62	48	
Age							
<60	91	56	35	**0.028**	43	48	**0.015**
≥60	147	69	78		93	54	
Pathology differentiation							
High (Middle or High)	175	96	79	0.229	89	86	**0.001**
Low (No or Low)	63	29	34		47	16	
T classification							
T1-2	44	27	17	0.193	17	27	**0.006**
T3-4	194	98	96		119	75	
N classification							
N0	101	67	34	**0.0002**	50	51	**0.041**
N1-2	137	58	79		86	51	
Metastasis							
No	231	122	109	0.892	133	98	0.698
Yes	7	3	4		3	4	
TNM stage							
I-IIA	96	65	31	**0.0001**	47	49	**0.036**
IIB-IV	142	60	82		89	53	
Vascular invasion							
No	172	97	75	0.053	97	75	0.707
Yes	66	28	38		39	27	
Neural invasion							
No	152	88	64	**0.027**	85	67	0.613
Yes	86	37	49		51	35	

Patients were staged in accordance with the 8^th^ Edition of the AJCC Cancer’s’ TNM Classification. Chi-square test, numbers in bold indicate significant p-values (p<0.05). H: high, L: low.

**Table 2:** Univariate and multivariate Univariate cox regression models for overall survival in PAAD patients (n = 238).

**Characteristics**	**Univariate analysis**			**Multivariate analysis**		
	**HR**	**95%CI**	**P value**	**HR**	**95%CI**	**P value**
Age	1.670	1.101-2.534	**0.016**	1.398	1.033-2.420	0.131
TNM stage	1.149	0.769-1.717	0.498	0.395	0.101-2.791	0.272
T classification	1.128	0.698-1.823	0.622	0.990	0.646-1.905	0.971
N classification	1.240	0.834-1.844	0.287	2.211	0.358-9.151	0.333
Metastasis	0.956	0.348-2.621	**0.029**	1.556	0.320-3.324	0.468
SCAMP1	1.565	1.068-2.294	**0.022**	1.654	1.005-2.266	**0.017**
SCAMP5	0564	0.380-0.838	**0.005**	0.531	0.377-0.872	**0.004**

Numbers in bold indicate significant p-values (p<0.05).

### SCAMP correlation analysis

**Figure 5:**
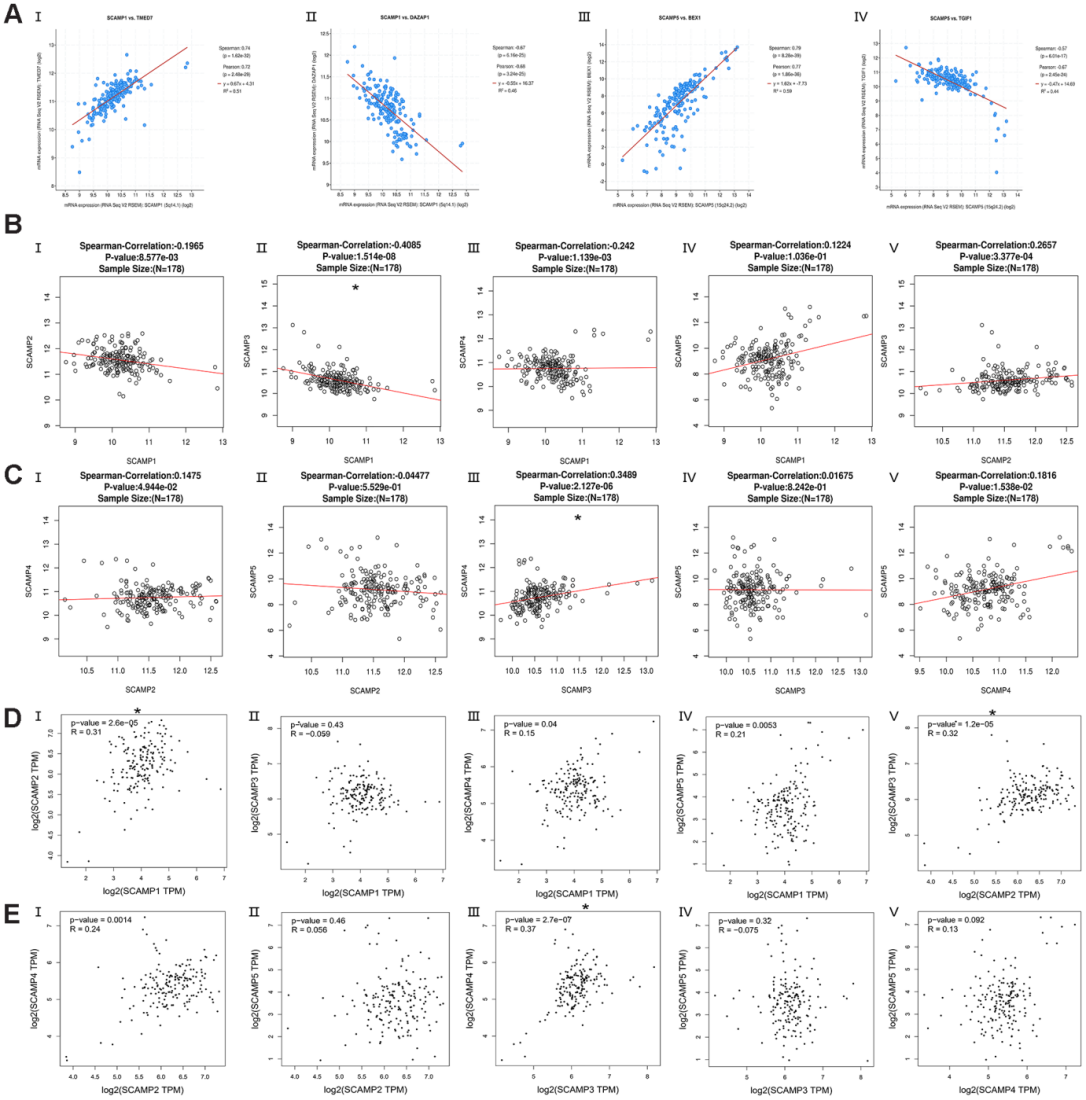
**Co-expressed genes of SCAMP1, 5, and correction between SCAMP1- 5 in PAAD (cBioPortal for Cancer Genomics, LinkedOmics, and GEPIA).** (**A**) Co-expressed genes (top-1) of SCAMP1, 5 in PAAD using the cBioPortal for Cancer Genomics. (I: TMED7, II: DAZAP1, III: BEX1, IV: TGIF1) (**B**, **C**) Correction between SCAMP1- 5 in PAAD using LinkedOmics. (B: I: SCAMP1 vs 2, II: SCAMP1 vs 3 III: SCAMP1 vs 4, IV: SCAMP1 vs 5, V: SCAMP2 vs 3; C: I: SCAMP2 vs 4, II: SCAMP2 vs 5 III: SCAMP3 vs 4, IV: SCAMP3 vs 5, V: SCAMP4 vs 5). (**D**, **E**) Correction between SCAMP1- 5 in PAAD using GEPIA. (R: Spearman correlation analysis; B: I: SCAMP1 vs 2, II: SCAMP1 vs 3 III: SCAMP1 vs 4, IV: SCAMP1 vs 5, V: SCAMP2 vs 3; C: I: SCAMP2 vs 4, II: SCAMP2 vs 5 III: SCAMP3 vs 4, IV: SCAMP3 vs 5, V: SCAMP4 vs 5) * Spearman correlation >0.3 or Spearman correlation< -0.3 and p< 0.05.

The cBioPortal for Cancer Genomics was used to analyze the co-expression of SCAMPs1, 5 from 179 PAAD tumors. The most significant genes that correlated with SCAMPs 1 and 5 (Figure 5A) were TMED7 (positive Spearman’s Correlation=0.742 p<0.001), DAZAP1 (negative Spearman’s Correlation=-0.673 p<0.001) and BEX1 (positive Spearman’s Correlation= 0.786 p<0.001) and TGIF1 (negative Spearman’s Correlation= -0.572 p<0.001). Other identified genes are summarized in Supplementary Table 1. Using the LinkedOmics database, SCAMP1 was found to negatively correlate with SCAMP 3 (spearman correlation: -0.4085, p < 0.01), whilst SCAMP3 positively correlated with SCAMP4 (spearman correlation: 0. 3489, p < 0.0001) (Figure 5B, 5C). These data were verified in the GEPIA datasets in which SCAMP1 correlated with SCAMP2 (R: 0.31, p < 0.05), whilst SCAMP3 correlated with SCAMP4 (R: 0.37, p < 0.05), (Figure 5D, 5E) in PAAD samples.

### Functional enrichment analysis of SCAMPs 1, 4 and 5

**Figure 6:**
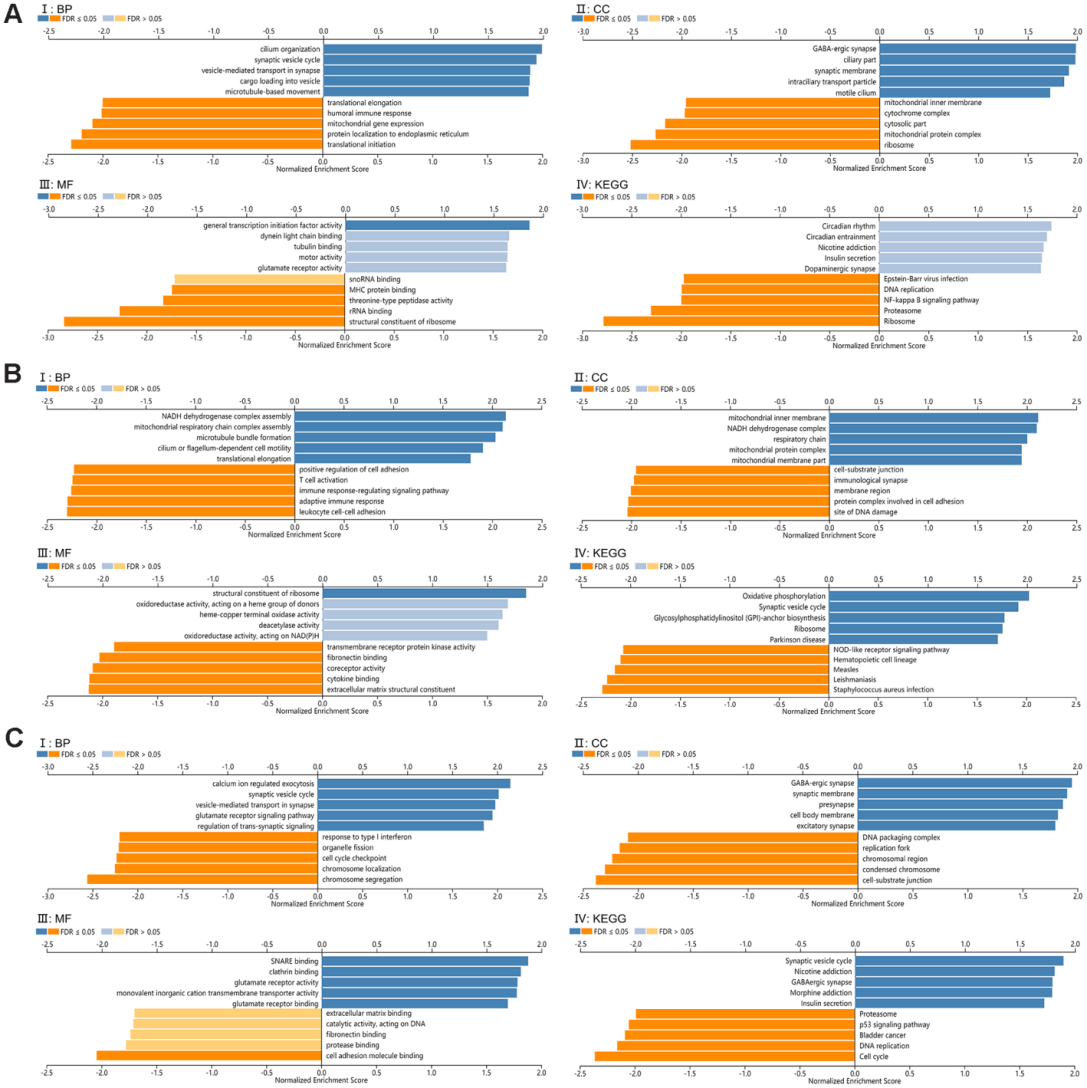
**Functional enrichment analysis for SCAMP1, 4, 5.** (**A**–**C**) Gene set enrichment analysis (GSEA) GO and KEGG pathway analysis for SCAMP1(**A**), 4(**B**), 5(**C**) respectively, (CC): Cellular components. (BP): Biological processes. (MF): Molecular functions. (KEGG): KEGG pathway analysis. (I: BP, II: CC, III: MF, IV: KEGG).

GO and KEGG pathway analysis of SCAMPs 1, 4 and 5 were performed to shed further light on their biological functions. GO term analysis in GSEA showed that the SCAMPs associated with the ribosomes (SCAMPs 1-4) and condensed chromosomes (SCAMP5) were they participate translational initiation (SCAMPS 1 and 4) or chromosome segregation (SCAMP 5). Furthermore, the SCAMPs were shown to participate in the structural constituent of ribosomes (SCAMPS 1 and 4) and cell adhesion molecule binding (SCAMP5) (Figure 6A–6C). KEGG pathway analysis showed enrichment in the ribosomes (SCAMPS 1 and 4) and cell cycle components (SCAMP 5) (Figure 6A–6C).

### Establishment and analysis of the PPI/ functional network

**Figure 7:**
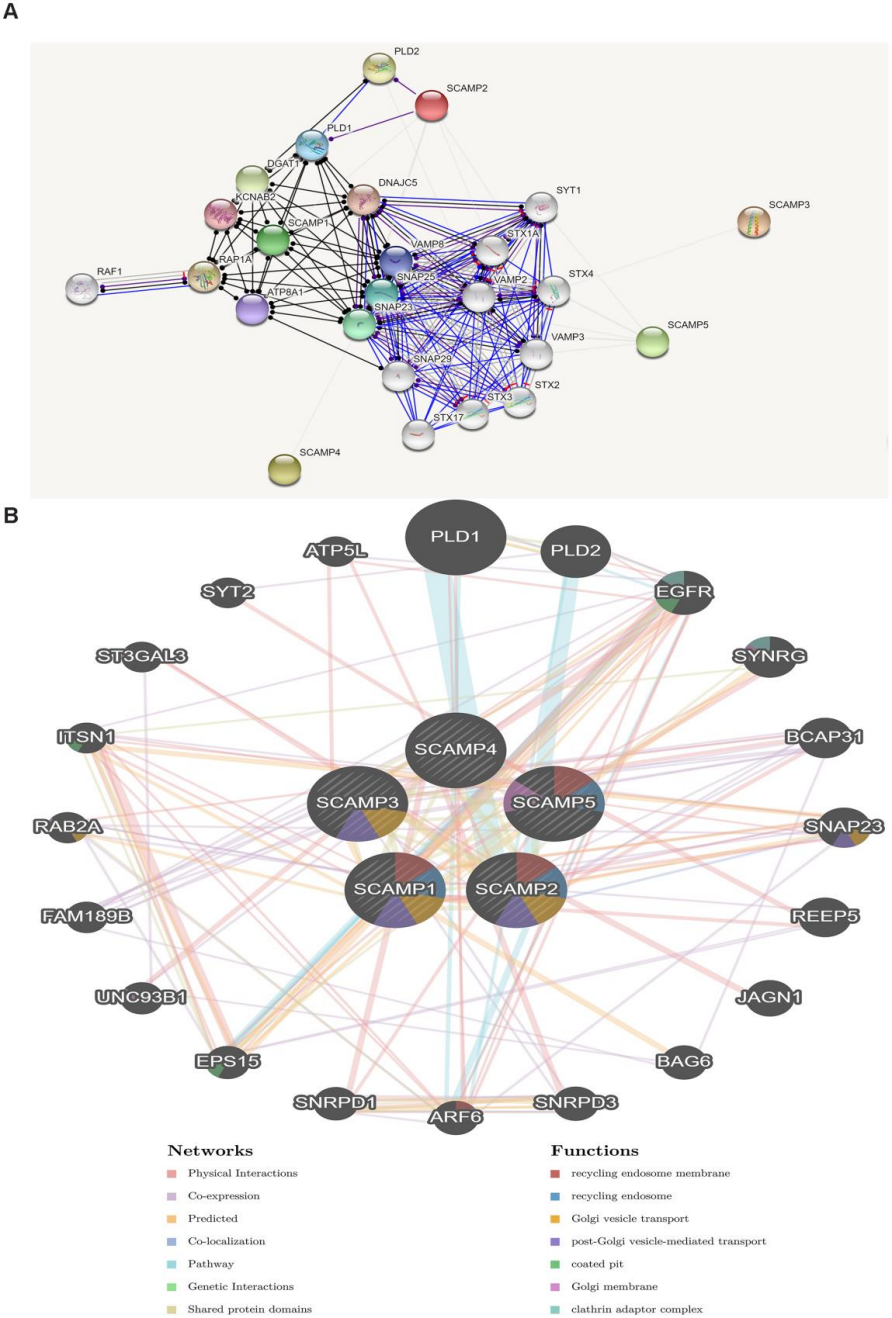
**Protein-protein interaction network of SCAMPs (STRING, GeneMANIA).** (**A**) Functional protein association networks of SCAMPs. Line shape indicated the predicted mode of molecular action. (**B**) Networks between predicted genes and SCAMP1-5. Different colors of the network edge indicate the bioinformatics methods applied: co-expression, website prediction, pathway, physical interactions and co-localization. Different colors for the network nodes indicate the biological functions of enrichment genes.

We firstly constructed PPI network of SCAMPs family by the predicted mode of molecular action, and constructed a two-layered model to reveal the regulatory networks of the SCAMPs in PAAD using GeneMania. The outer layer included genes co-expressed and interacting with PLD1, ATP5L, PLD2, SYT2, EGFR, ST3GAL3, SYNRG, BCAP31, ITSN1, RAB2A, SNAP23, FAM189B, REEP5, UNC93B1, JAGN1, EPS15, BAG6, SNRPD1, ARF6, and SNRPD3 (Figure 7).

## DISCUSSION

The dysregulation of SCAMPs occurs frequently in an array of cancers [10–16]. This study explored the differential expression and prognostic value of SCAMPs in PAAD in an attempt to improve PAAD treatment and diagnostic accuracy.

Emerging studies suggest that the dysregulation of SCAMP1 is related to the occurrence and progression of various tumors, including pancreatic cancer, gallbladder cancer, cervical cancer and breast cancer [10–12]. The downregulation of SCAMP1 suppresses the migration and invasion of tumor cells [11, 17]. SCAMP1 upregulates VEGF secretion that is required for nutrient support for newly formed blood vessels [18]. SCAMP1 also suppresses the malignant proliferation of glioma cells through the miR-499a-5p/LMX1A/NLRC5 axis, highlighting SCAMP1 as an oncogene [19]. SCAMP1 is a key cellular regulator of endocytic and secretory pathways through its dual role in the stimulation of dilation and blocking fusion pores, which downregulates bulk exocytosis [20]. In this study, the aforementioned cancer databases demonstrated that SCAMP1 expression is elevated in PAAD vs. healthy tissue. These findings were confirmed following the assessment of SCAMP1 levels in a range of *in vitro* PAAD culture systems, in which SCAMP1 was significantly elevated compared to non-cancerous pancreatic cell lines. Combining GEPIA and HPA datasets with our clinical data revealed that SCAMP1 provides strong prognostic value in PAAD patients. Upregulated SCAMP1 significantly correlated with poor OS.

We found that the expression of SCAMPs 2 and 3 in PAAD samples were higher than normal pancreatic samples. SCAMP 2 and 3 expression were also elevated in human PAAD cell lines according to CCLE and EMBL-EBI databases. No positive correlation was observed between SCAMPs 2 and 3 and OS in PAAD. To-date the role of SCAMP2 in cancer is poorly defined. SCAMP2 also colocalized with fusion sites and enhanced granule exocytosis mediated through E peptide–containing domains that inhibit exocytosis [7]. Together, these data suggest that SCAMP2 promotes NHE5 transport through recycling endosomes and enhances its cell-surface targeting in an Arf6-dependent manner. NHE5 is a Na^+^/H^+^ exchanger enriched in brain tissue [21]. A correlation between the expression of a basic/hydrophobic peptide segment within SCAMP2: CWYRPIYKAFR that interacts with PI (4, 5) P2) and inhibits exocytosis, particularly by SC2-R204A exists. A common electrostatic interaction is known to occur between PI (4, 5) P2 and the E peptides of SCAMPs 1 and 2 that regulates their interaction within the membrane interface during exocytosis. Similar interactions involving other SCAMPs may also exist but as yet remain undefined [22]. SCAMP2 also regulates exocytosis through other methods [23].

It was recently suggested that SCAMP3 acts as a prognostic biomarker and treatment target in HCC, the silencing of which suppresses HCC proliferation and cell cycle progression [16]. SCAMP3 inhibits endocytosis and receptor degradation through its ability to inhibit the ubiquitination of ESCRTs (Endosomal Sorting Complex Required for Transports) [24]. SCAMP3 positively influences the sorting and budding of intraluminal vesicles controlling multivesicular endosome biogenesis [25]. Multivesicular bodies (MVBs) regulate cell cycle progression and tumor biogenesis. Chmp1A, an ESCRT protein, inhibits cancer cell proliferation, invasion and signaling activity via MVB formation [26], highlighting the potential role of SCAMP3 during cancer development. In our analysis, SCAMP3 expression was minimally associated with the OS of PAAD patients.

The enhanced phosphorylation of PKC by SCAMP-4 alters vesicular transport [27]. SCAMP5 enhances the secretion of calcium-regulated signal peptide-containing cytokine (CL5) but not IL-1β in MCF-7 epithelial cells [28]. SCAMP5 co-operates with SNAREs to enhance cytokine exocytosis through a process mediated by enhanced Ca^2+^ influx [28]. The role of SCAMPs 4-5 in tumor cells are less well-studied. Our data suggest that SCAMP4 is overexpressed in PAAD vs normal tissues, whilst SCAMP5 shows an opposite phenotype. Surprisingly, elevated levels of SCAMP5 correlate with an improved OS in PAAD patients. This highlights the tumor suppressive role of SCAMP5 in PAAD cells.

We next assessed the utility of SCAMPs as prognostic indicators of PAAD, to reveal important information on the molecular roles of these trafficking proteins in the development and progression of PAAD. We found that SCAMPs 1 and 5 showed dysregulated expression in PAAD tumors and play an important role in PAAD tumorigenesis, serving as molecular markers for those at an elevated risk of PAAD tumorigenesis. These data also highlighted SCAMPs 1 and 5 as potential targets for PAAD treatment, the regulation of which could improve PAAD survival and prognosis. SCAMP 1, 5 expression were significantly associated with age, N classification and TNM-stage. Enrichment analysis suggested that the functional network of SCAMPs were involved in the structural constituents of ribosomes and cell adhesion molecule binding. We further highlighted key gene members in the functional activity of the SCAMPs including PLD1, ATP5L, PLD2, SYT2, EGFR, ST3GAL3, SYNRG, BCAP31, ITSN1, RAB2A, SNAP23, FAM189B, REEP5, UNC93B1, JAGN1, EPS15, BAG6, SNRPD1, ARF6, and SNRPD3. Amongst them, PLD1, EGFR, ST3GAL3 and ARF6 mediate the progression of pancreatic cancer [29–33].

Overall, this study reveals that all SCAMP family members are differentially expressed between PAAD and normal tissues, and that SCAMPs 1, 5 may act as predictors for the diagnosis and prognosis of PAAD. SCAMPs therefore represent potential therapeutic targets for PAAD and mediate its occurrence and progression of PAAD, as in other cancers. This study still had some limitations: Firstly, our SCAMP data were obtained from public databases and verification queues only included SCAMP protein expression levels without confirmation *in vitro or in vivo*. Secondly, diagnosis and prognosis require extensive external verification of the clinical sample data and our diagnosis model is from tissue samples without blood or exosome samples. Thirdly, specific functional regulatory networks cannot be built without amount experiments by only bioinformatics analysis. Further studies are therefore required to verify the role played by SCAMPs in PAAD.

## MATERIALS AND METHODS

### Patients and tissue specimens

Pancreatic adenocarcinoma tissues were obtained from patients admitted to the Northern Jiangsu People’s Hospital who had surgical abscission treatment from October 2015 to October 2019. Patients were individually diagnosed by 2 pathologists, had not received chemotherapy or radiation therapy prior to surgical procedures. A total of 238 cancerous and 117 noncancerous paraffin-embedded specimens were used for immunohistochemistry analysis.

### Real-time quantitative PCR and western blot assay

Shanghai Institute of Nutrition and Health in Shanghai, China provided the human PAAD cell lines PANC-1, SW1990, AsPC-1, and normal HPDE cell (Human pancreatic ductal cell). A one percent strength streptomycin-penicillin along with fetal bovine serum of ten percent strength was part of the medium in which the cells were cultured at a temperature of 37° C and five percent carbon dioxide- fed humidified incubator.

The Trizol reagent (from Invitrogen, (China) was used to extract the total ribonucleic acid while the PT-PCR Kit sourced from Vazyme (China) was used to reverse transcribe the samples. The primers used in this study were: SCAMP1- forward: 5′-TTCGACAGTAACCCGTTTGC-3′; SCAMP1- reverse: 5′-ATTAGGCATCTTCACACCGC-3′; SCAMP2- forward: 5′-CAGAGATCCCTGCCGACTAC-3′; SCAMP2- reverse: 5′-CAGGCAAGCAGGTTCAGAAA-3′; SCAMP3- forward: 5′-ATCCACTCCTTATACCGCCG-3′; SCAMP3- reverse: 5′-GAGAAGACACCAGCAGCAAA-3′; SCAMP4- forward: 5′-TTCCGGCCTGTCTACAAGG-3′; SCAMP4- reverse: 5′-AACTGGGCTCCGAAGATGAA-3′;SCAMP5- forward: 5′-ACCAAGACTTCGAGGCAGAT-3′;SCAMP5- reverse: 5′-CGCTGTTCAACATCCAGAGG-3′; C3H6O3 (Glyceraldehyde) -3-G6PD_N (phosphate dehydrogenase): forward: 5′-GGTGAAGGTCGGAGTCAACG-3′; reverse: 5′-CAAAGTTGTCATGGATGACC-3′. GAPDH Ct was the value to which each Ct value expression was normalized to determine relative expressions. RT-qPCRs were performed as the MIQE (Minimum Information for Publication of Quantitative Real-Time PCR Experiments) guidelines [34].

RIPA (from Solarbio (China) was used to lyse the cells. The radioimmunoprecipitation assay contained a protease inhibitor maintained on ice for thirty minutes. A five percent mixture of bovine serum albumin from Invitrogen (China) was used to block the membranes and incubation was done overnight at 4° C. Subsequently, for a duration of 1 h, incubation was conducted using horseradish peroxidase (HRP)-conjugated goat anti-mouse or goat anti-rabbit sourced from Cell Signaling Technology, China. SCAMP1, SCAMP2, SCAMP3, SCAMP4 and SCAMP5 antibodies sourced from proteintech, China was used to perform western blot assay with anti-GAPDH antibody (Abcam) as a loading control. Data was analyzed by GraphPad Prism 8.0 (GraphPad Software Inc., San Diego, CA) and presented as mean ± SD. One-way analysis of variance (ANOVA) followed Tukey’s test was employed to compare differences among multiple groups. P < 0.05 was indicated as statistically significant.

### Immunohistochemistry

The sample was blocked and incubated with the SCAMP1, 5 Ab (1: 50) for 2 hours at 23° C, and an HRP conjugated goat anti-rabbit Ab was used as the secondary probe. 2 pathologists, independent of each other and blinded by the patients’ clinical data gave the evaluation of immunohistochemical staining. SCAMP1, 5 expression levels were classified by the semi-quantitative method that combines the intensity of the staining as well as the percentage of cells that stained positive. [35, 36]

### GEPIA datasets

GEPIA is an online analysis tool that provides a python package for the rapid analysis and retrieval of data based on TCGA and GTEx datasets. The database provides an interactive and customizable function including differential expression analysis, profiling plots, correlation analysis, survival analysis, gene analysis, and dimensionality reduction [37]. We used log-rank tests (Mantel–Cox tests) for hypothesis evaluation and selected the Cox proportional hazard ratio and 95% confidence interval for survival analysis.

### LinkedOmics datasets

LinkedOmics can compare multi-omic cancer datasets across an array of tumor types (32 cancers and 11,158 patients) from the TCGA project and proteomics data from the CPTAC. LinkedOmics contains three key analysis modules: namely LinkFinder, LinkCompare, and LinkInterpreter, and displays data in the form of volcano plots, heat maps, or scatter plots [36]. The LinkInterpreter module builds statistical plots for individual genes and performs pathway and functional network analyses of differentially expressed genes (DEGs). This comprehensive, flexible and interactive functional category database provides an online gene set analysis toolkit (WebGestalt) which was applied in this study [38, 39]. LinkedOmics was used to sign and rank the data from the LinkFinder, which was selected for GSEA to perform GO (BP, CC and MF), and KEGG analysis. We used non-parametric analysis and Pearson Correlation tests to obtain our data. Criterion were ranked with an FDR < 0.05. A total of 500 simulations were performed.

### CCLE datasets

The CCLE is an amalgamation of copy number data, gene expression analysis, and parallel sequencing assessments from 947 human cancer cell lines formed by the Broad and Novartis Institutes for Biomedical Research and the Genomics Institute of the Novartis Research Foundation. The CCLE can be used for the assessment of cell targets, gene variants, small-molecules and therapeutics, permitting the identification of novel marker-driven cancer dependencies. The CCLE datasets and their accompanying public data portals provide a resource to promote cancer research using model *in vitro* cancer cell lines [40–43]. SCAMP expression in cancer cell lines can be verified using the CCLE dataset.

### EMBL-EBI (expression atlas) dataset

The expression Atlas was used to verify SCAMP expression in the PAAD cell lines. EMBL-EBI is a continually updated database that provides gene and protein expression data in an array of species and contexts, including tissue development, diseases and cell types. The expression Atlas includes 1101 studies on human microarrays and RNA-sequencing data from Blueprint, PCAWG, ENCODE, GTEx and HipSci databases [44, 45].

### HPA datasets

HPA acts as a roadmap for all human protein-protein interactions through its integration of antibody-based imaging, mass spectrometry-based proteomics, transcriptomics and systems biology, thus providing an interactive web-based tool to explore gene expression and survival in 17 cancer types. The Tissue Atlas and Human Pathology Atlas characterize the expression and localization of human proteins in various tissues and organs according to RNA-seq, immunohistochemistry of tissue microarrays and transcriptomes using data from ≥ 8000 patients [46–48].

### cBioPortal for cancer genomics

The cBioPortal for Cancer Genomics is an open platform that permits the interactive exploration of multidimensional cancer genomic datasets. The cBioPortal allows researchers to convert complex genomic data into visual biological insights and clinical applications including somatic mutations, DNA copy-number alterations (CNAs), mRNA and microRNA (miRNA) expression, DNA methylation, protein expression and phosphoprotein levels [49, 50].

### STRING and GeneMANIA

Search Tool for the Retrieval of Interacting Genes (STRING; http://string-db.org) (version 11.0) database can provide the analysis of functional interactions between proteins, and provide insights for the research on the mechanism of disease occurrence or progression [51]. Co-expressed genes that co-localize or interact either directly or with the targets of SCAMPs were identified using GeneMania. The database encompasses data from the GEO, physical and genetic interaction data from BioGRID and predicted protein interactions based on orthology from I2D. Pathways and molecular interaction data were derived from the Pathway Commons [52]. SCAMPs and their functional networks in PAAD were analyzed using this STRING and GeneMania.

## Supplementary Material

Supplementary Figure 1

Supplementary Table 1
