# Supplementary material for: Expression and prognostic analyses of SCAMPs in pancreatic adenocarcinoma
Source: Aging (Albany NY). 2021 Jan 20;13(3):4096–114. doi: 10.18632/aging.202377 (PMC7906166; doi:10.18632/aging.202377)
Supplement: Supplementary Figure 1 [file aging-13-202377-s001.pdf]

SUPPLEMENTARY FIGURE

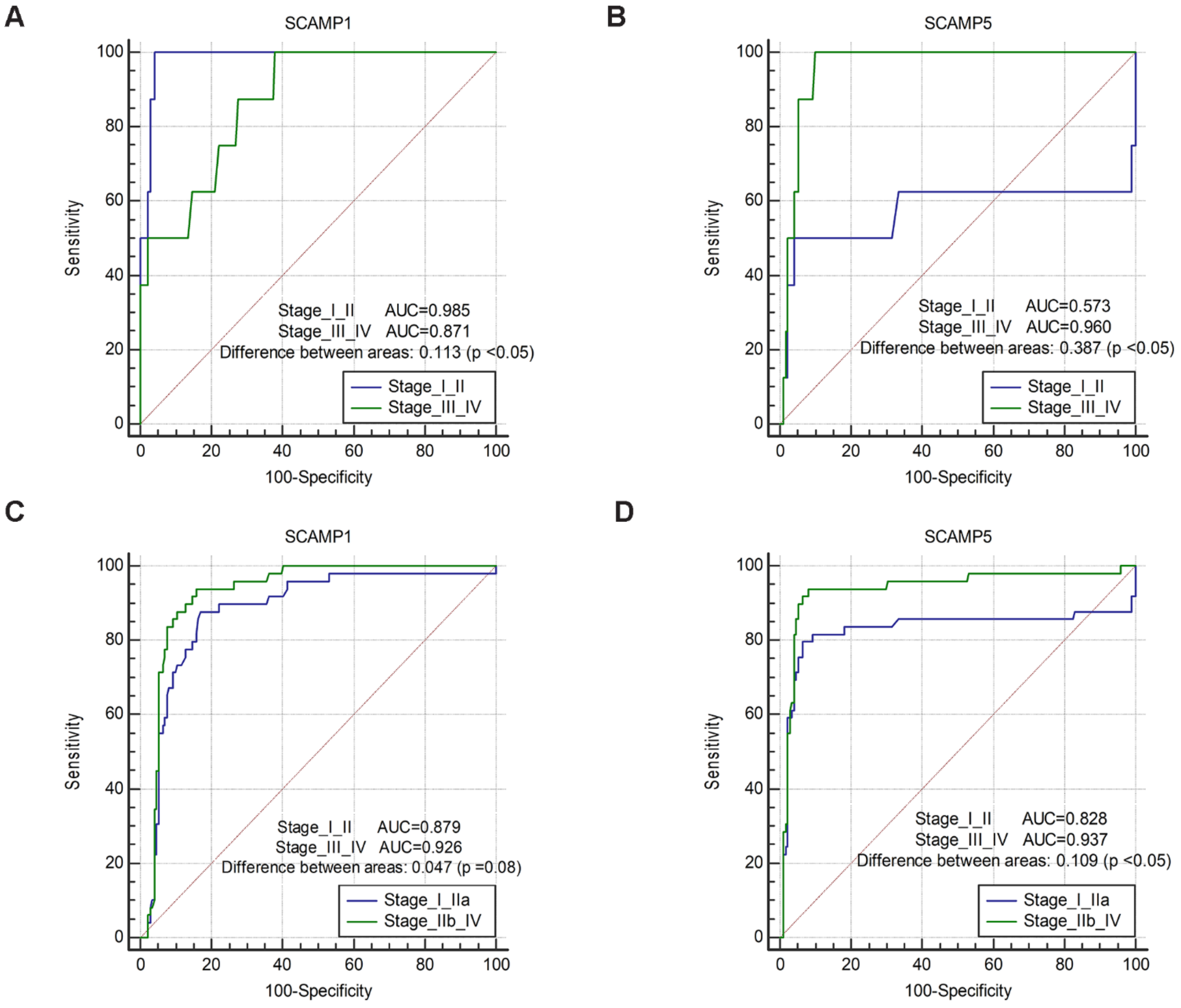

Supplementary Figure 1. The difference in SCAMP1 and SCAMP5 between early stage and late stages as diagnosis markers. (**A**, **B**) The significant difference in SCAMP1($p < 0.05$) and SCAMP5 ($p < 0.05$) between early stage (Stage I + II) and late stages (Stage III + IV) as diagnosis markers. (**C**, **D**) The difference in SCAMP1 ($p > 0.05$) (**C**) and SCAMP5 ($p < 0.05$) (**D**) between early stages (Stage I + II a) and late stages (Stage II b + III + IV). The Area Under the Curve (AUC) metrics are also provided for SCAMP1 and 5 to predict diagnosis in PAAD by Medcalc.
