# Supplementary material for: Expression and prognostic analyses of SCAMPs in pancreatic adenocarcinoma
Source: Aging (Albany NY). 2021 Jan 20;13(3):4096–114. doi: 10.18632/aging.202377 (PMC7906166; doi:10.18632/aging.202377)
Supplement: Supplementary Table 1 [file aging-13-202377-s002.pdf]

SUPPLEMENTARY TABLE

Supplementary Table 1. The most significant genes-Top 10 correlated with SCAMPs 1 and 5 by cBioPortal.

	SCAMP1				SCAMP5		
	**Correlated gene**	**Spearman's correlation**	**p-Value**		**Correlated gene**	**Spearman's correlation**	**p-Value**
POSITIVE	TMED7	0.741659	1.62E-32	POSITIVE	BEX1	0.785973	8.28E-39
	COL4A3BP	0.736721	6.78E-32		OGDHL	0.775737	3.15E-37
	PJA2	0.717279	1.41E-29		RUNDC3A	0.755899	2.16E-34
	PPM1A	0.717126	1.46E-29		CHGA	0.751231	9.19E-34
	TNPO1	0.716946	1.53E-29		AMER3	0.749632	1.50E-33
	UBL3	0.710301	8.57E-29		TMEM63C	0.744419	7.18E-33
	TMEM167A	0.698749	1.52E-27		KCNJ11	0.743529	9.34E-33
	C5ORF24	0.687555	2.17E-26		ABCC8	0.741569	1.66E-32
	BDP1	0.684726	4.18E-26		SCGN	0.741037	1.94E-32
	AKAP11	0.682395	7.12E-26		DUSP26	0.740452	2.31E-32
NEGATIVE	DAZAP1	-0.67272	6.16E-25	NEGATIVE	TGIF1	-0.57208	6.01E-17
	CYBC1	-0.65368	3.43E-23		NDE1	-0.54588	2.73E-15
	PFN1	-0.64466	2.09E-22		HRH1	-0.53127	1.99E-14
	BCL2L12	-0.634	1.63E-21		MET	-0.52996	2.37E-14
	GALK1	-0.6296	3.73E-21		FAM83D	-0.50956	3.23E-13
	IKBKG	-0.62792	5.09E-21		ECT2	-0.50923	3.36E-13
	RHOG	-0.62117	1.75E-20		TEAD3	-0.50606	4.96E-13
	RPS6KB2	-0.61867	2.74E-20		CDC25C	-0.50488	5.73E-13
	ALDH16A1	-0.61025	1.21E-19		TFAP2A	-0.49735	1.42E-12
	RPLP1	-0.60608	2.48E-19		TRIM59	-0.49657	1.56E-12

The most significant genes-Top 10 correlated with SCAMPs 1 and 5 by cBioPortal.
